# Preoperative C-reactive protein/albumin ratio and mortality of off-pump coronary artery bypass graft

**DOI:** 10.3389/fcvm.2024.1354816

**Published:** 2024-03-15

**Authors:** Ah Ran Oh, Ji-Hye Kwon, Jungchan Park, Jeong-Jin Min, Jong-Hwan Lee, Seung Yeon Yoo, Dong Jae Lee, Wooksung Kim, Hyun Sung Cho, Chung Su Kim, Sangmin Maria Lee

**Affiliations:** ^1^ Department of Anesthesiology and Pain Medicine, Samsung Medical Center, Sungkyunkwan University School of Medicine, Seoul, Republic of Korea; ^2^ Department of Anesthesiology and Pain Medicine, Kangwon National University Hospital, Chuncheon, Republic of Korea; ^3^ Department of Thoracic and Cardiovascular Surgery, Samsung Medical Center, Sungkyunkwan University School of Medicine, Seoul, Republic of Korea

**Keywords:** albumin, biomarker, coronary artery bypass, C-reactive protein, mortality

## Abstract

**Background:**

We sought to investigate the prognostic value of preoperative C-reactive protein (CRP)-to-albumin ratio (CAR) for the prediction of mortality in patients undergoing off-pump coronary artery bypass grafting (OPCAB).

**Methods:**

From January 2010 to August 2016, adult patients undergoing OPCAB were analyzed retrospectively. In a total of 2,082 patients, preoperative inflammatory markers including CAR, CRP, neutrophil-to-lymphocyte ratio, and platelet-to-lymphocyte ratio were recorded. Receiver operating characteristic (ROC) curves were used to determine the optimal threshold and compare the predictive values of the markers. The patients were divided into two groups according to the cut-off value of CAR, and then the outcomes were compared. The primary end point was 1-year mortality.

**Results:**

During the 1-year follow-up period, 25 patients (1.2%) died after OPCAB. The area under the curve of CAR for 1-year mortality was 0.767, which was significantly higher than other inflammatory markers. According to the calculated cut-off value of 1.326, the patients were divided into two groups: 1,580 (75.9%) patients were placed in the low CAR group vs. 502 (24.1%) patients in the high CAR group. After adjustment with inverse probability weighting, high CAR was significantly associated with increased risk of 1-year mortality after OPCAB (Hazard ratio, 5.01; 95% Confidence interval, 2.01–12.50; *p* < 0.001).

**Conclusions:**

In this study, we demonstrated that preoperative CAR was associated with 1-year mortality following OPCAB. Compared to previous inflammatory markers, CAR may offer superior predictive power for mortality in patients undergoing OPCAB. For validation of our findings, further prospective studies are needed.

## Introduction

Coronary artery bypass grafting (CABG) is the most effective treatment for multivessel coronary artery disease (CAD), and the most frequently performed open cardiac surgery worldwide (1, 2). Despite improvements in perioperative care and operative techniques, CABG remains a complex and high-risk procedure with significant morbidity and mortality (3). Inflammation plays a key role in the pathogenesis and progression of CAD (4, 5). When performing surgical revascularization, potent inflammatory mediators are released, adversely affecting patient status (6, 7). In this context, several studies have evaluated the association between inflammatory markers and adverse outcomes after CABG in terms of prognostic value (8–11).

Recently, C-reactive protein (CRP)/albumin ratio (CAR) has been used as a novel indicator of inflammation and a prognostic marker for patients with severe critical illness (12, 13). High levels of CRP indicate an active inflammation process, which is associated with increased risk of cardiac events in patients with CAD (14). Albumin is inversely related to inflammation and low albumin level also has been recognized as a risk factor for cardiovascular disease (15). Therefore, the use of both CRP and albumin together may reflect inflammatory status better than either marker alone in CAD. Previous studies have confirmed that CAR is superior to CRP or albumin alone for assessing the severity and prognosis of patients with CAD (16, 17). However, the relationship between CAR and mortality has not been evaluated in patients undergoing CABG. Therefore, we sought to determine whether preoperative CAR is a predictor of postoperative mortality after off-pump CABG (OPCAB). The results of this study may help to identify patients at high risk for mortality after OPCAB, allowing for earlier interventions and improved outcomes.

## Methods

This retrospective observational cohort study was approved by the Institutional Review Board at Samsung Medical Center (reference number 2022-05-087). It adhered to the Declaration of Helsinki and followed the Strengthening the Reporting of Observational Studies in Epidemiology (STROBE) guidelines. Due to the low risk to participants and the retrospective nature of the study, the requirement for written consent from individual patients was waived.

### Study population and data collection

For this study, we reviewed the records of consecutive adult patients who underwent OPCAB at our hospital between January 2010 and August 2016. In the case of re-operation, only the first operation was included in analysis, and patients without preoperative CRP or albumin were excluded. We included only the initial operation was to maintain uniformity in our dataset and reduce the complexity associated with repeated surgeries. Multiple surgeries in the same individual may introduce additional variables, such as variations in postoperative care, recovery trajectories, and underlying health conditions, which could complicate the analysis.It was a single center cohort of multiple surgeons with different OPCAB technique. We created this cohort by accessing an electronic archive system containing information on over four million patients, including more than 1.2 billion laboratory results and 300 million prescriptions. The data were obtained in de-identified form from our electronic system, the “Clinical Data Warehouse DARWIN-C” of Samsung Medical Center. All relevant information such as demographic, laboratory, and outcome data were organized by independent investigators who were not involved in this study. The laboratory tests were conducted within a period of 6 months prior to the surgery. This timeframe aligns with the institution's protocol, which deems blood lab test results within six months as an acceptable and practical reflection of patients' baseline health status.To calculate the CAR value, the CRP (mg/L) level was divided by the serum albumin (g/dl) level. The ratio of neutrophils to lymphocytes (NLR) was calculated by taking the number of neutrophils and dividing it by the number of lymphocytes. Similarly, the ratio of platelets to lymphocytes (PLR) was determined by dividing the number of platelets by the number of lymphocytes. Our electronic system regularly receives updates to the mortality data from the National Population Registry of Korea.

### Study endpoint

The primary endpoint of this study was all-cause mortality during 1-year follow-up. Secondary endpoints were the major cardiovascular and cerebrovascular events (MACCE) during 1-year follow-up and all-cause mortality during three and 5-year follow-ups. MACCE was defined as a composite of following outcomes: all-cause death, graft failure, coronary revascularization, myocardial infarction, and stroke.

### Statistical analysis

Continuous variables were presented as means with standard deviation or medians with interquartile range (IQR), and categorical variables were expressed as numbers with percentages. To compare the differences between the two groups, continuous variables were evaluated using *t*-test or the Mann–Whitney test, while categorical variables were assessed with the *χ*^2^ test or Fisher's exact test. To determine the best threshold for CAR, CRP, NLR, and PLR in predicting 1-year mortality, we used receiver operating characteristic (ROC) curve analysis and calculated Youden's index. To assess the predictive power of the markers, the values of the area under the curve (AUC) with 95% confidence interval (CI) were compared using DeLong's test (18). The patients were divided into low and high groups based on the calculated cut-off value of CAR. To minimize the impact of selection bias and confounding factors, we conducted inverse probability weighting (IPW) to adjust for differences in the baseline characteristics of the patients using all relevant variables shown in Table 1 (19). To calculate the weight for each patient, we estimated a probability that predicts the likelihood of being included in the high CAR group, and the weight for each patient was calculated as the inverse of the probability. A standardized mean difference of less than 10% was considered as an adequate balance between the two groups. To compare the mortality and MACCE, we used Cox regression analysis and weighted Cox proportional hazard analysis with IPW, and the results were presented as a hazard ratio (HR) and 95% CI. Kaplan–Meier curves were constructed for 1-year MACCE and compared with the log-rank test. Statistical analysis for this study was conducted using R 4.2.0 (Vienna, Austria; http://www.R-project.org/) and a *P*-value of less than 0.05 was considered statistically significant.

**Table 1:** Baseline characteristics according to 1-year mortality.

	Survivor (*N* = 2,057)	Non-survivor (*N* = 25)	*p-*value
C-reactive protein/albumin ratio	0.33 (0.12–1.21)	1.74 (0.82–5.15)	<0.001
Neutrophil/lymphocyte ratio	1.95 (1.46–2.73)	2.41 (1.86–2.84)	0.13
Platelet/lymphocyte ratio	7.07 (5.46–9.25)	7.88 (5.75–12.39)	0.13
C-reactive protein, mg/L	1.4 (0.5–4.9)	6.9 (3.1–17.5)	<0.001
Albumin, g/dl	4.2 (4.0–4.4)	3.9 (3.5–4.1)	<0.001
Neutrophil	58.8 (52.3–65.6)	61.4 (55.0–65.5)	0.61
Lymphocyte	30.0 (23.7–36.0)	26.5 (21.9–30.6)	0.05
Platelet, K/mcL	209 (175–247)	203 (175–241)	0.85
Age, years	63.2 (±10.0)	71.6 (±7.5)	<0.001
Male	1,601 (77.8)	20 (80.0)	0.99
Smoking	545 (26.5)	5 (20.0)	0.61
Body mass index	24.6 (±3.0)	24.3 (±3.6)	0.63
Hypertension	1,633 (79.4)	20 (80.0)	>0.99
Diabetes	937 (45.6)	9 (36.0)	0.45
Old myocardial infarction	183 (8.9)	3 (12.0)	0.85
Acute myocardial infarction	221 (10.7)	4 (16.0)	0.61
Ejection fraction	57.0 (±12.3)	50.0 (±15.9)	0.01
Previous coronary intervention			
Percutaneous intervention	360 (17.5)	1 (4.0)	0.13
Bypass grafting	7 (0.3)	0	>0.99
Previous disease			
Peripheral arterial occlusive disease	105 (5.1)	3 (12.0)	0.28
Chronic obstructive pulmonary disease	27 (1.3)	2 (8.0)	0.05
Stroke	257 (12.5)	6 (24.0)	0.16
Chronic kidney disease	112 (5.4)	5 (20.0)	0.01
Dialysis	58 (2.8)	4 (16.0)	0.001
Heart failure	29 (1.4)	0	>0.99
Valvular disease	10 (0.5)	0	>0.99
Aortic disease	15 (0.7)	0	>0.99
Drug use			
Statin	1,065 (51.8)	10 (40.0)	0.33
Antiplatelet	1,937 (94.2)	24 (96.0)	>0.99
Renin-angiotensin-aldosterone system inhibitor	649 (31.6)	11 (44.0)	0.27
Beta blocker	688 (33.4)	7 (28.0)	0.72
Calcium channel blocker	641 (31.2)	11 (44.0)	0.25
Operative variables			
Urgency operation	49 (2.4)	1 (4.0)	>0.99
Operative duration, min	269.1 (±68.7)	283.2 (±66.4)	0.31
Red blood cell transfusion, pack	2.2 (±1.5)	3.6 (±1.2)	<0.001

Values are *n* (%) or mean (±SD)/median (interquartile range).

## Results

From January 2010 to August 2016, a total of 2,082 adult patients who underwent OPCAB in our institution were enrolled in final analysis. During the 1-year follow-up period, 25 patients (1.2%) died after OPCAB. The baseline characteristics of the patients according to 1-year mortality are summarized in Table 1. The median values of CAR and CRP were significantly higher in the non-survivor group, but albumin was significantly lower.

**Figure 1:**
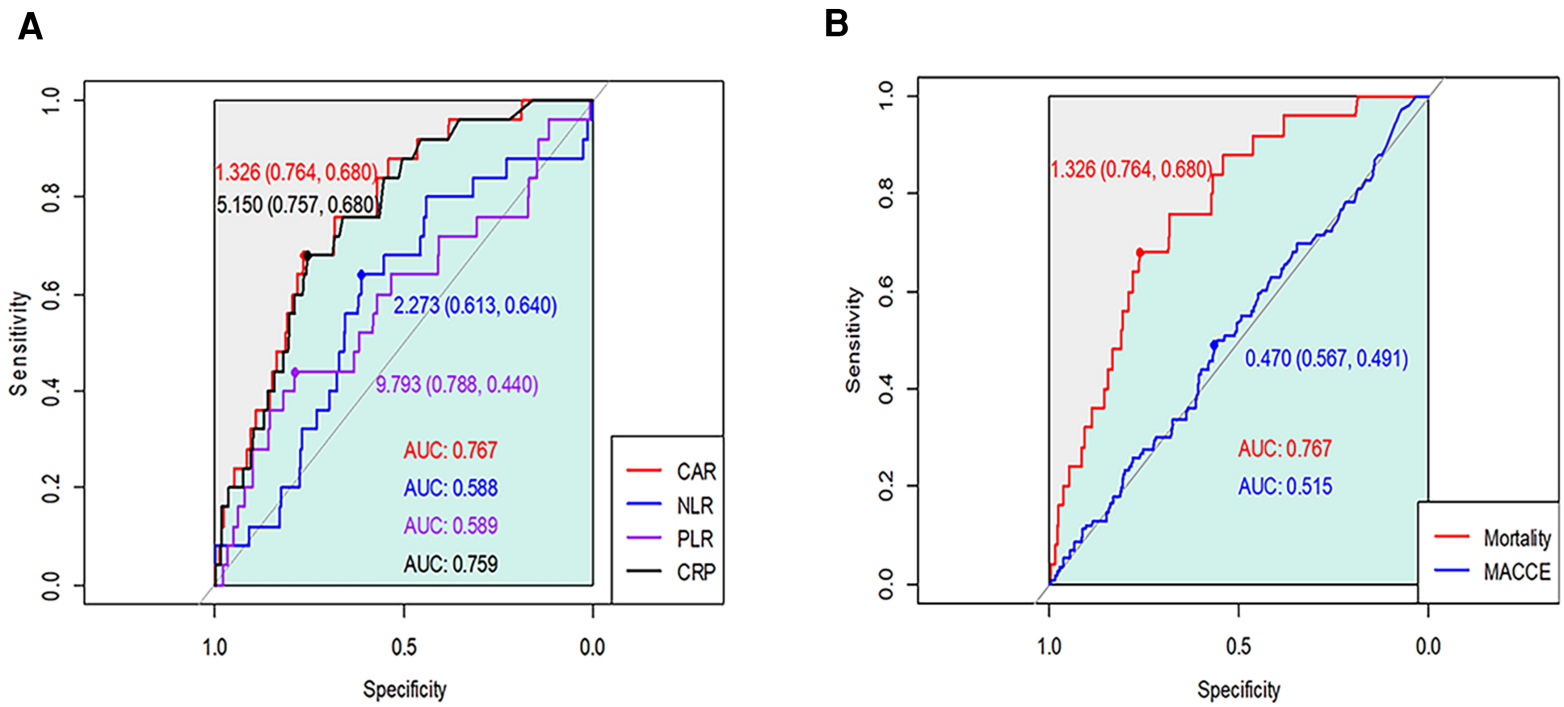
Receiver operating characteristic curves of CAR, CRP, NLR, and PLR for (**A**) 1-year mortality and (**B**) major cardiovascular and cerebrovascular events after OPCAB.

We generated the ROC curves of CAR, CRP, NLR, and PLR for the prediction of 1-year mortality, and the AUCs with 95% CI were 0.767 (0.684–0.850), 0.759 (0.675–0.842), 0.588 (0.475–0.701), and 0.589 (0.461–0.716), respectively (Figure 1A). On comparison of the AUC values, CAR showed significantly better predictive power than other inflammatory markers (*Z* = 2.86, *p* = 0.004 for CAR vs. CRP). Based on maximum Youden's index, the optimal threshold of CAR, CRP, NLR, and PLR for predicting 1-year mortality were 1.326, 5.150, 2.273, and 9.793, respectively.

**Figure 2:**
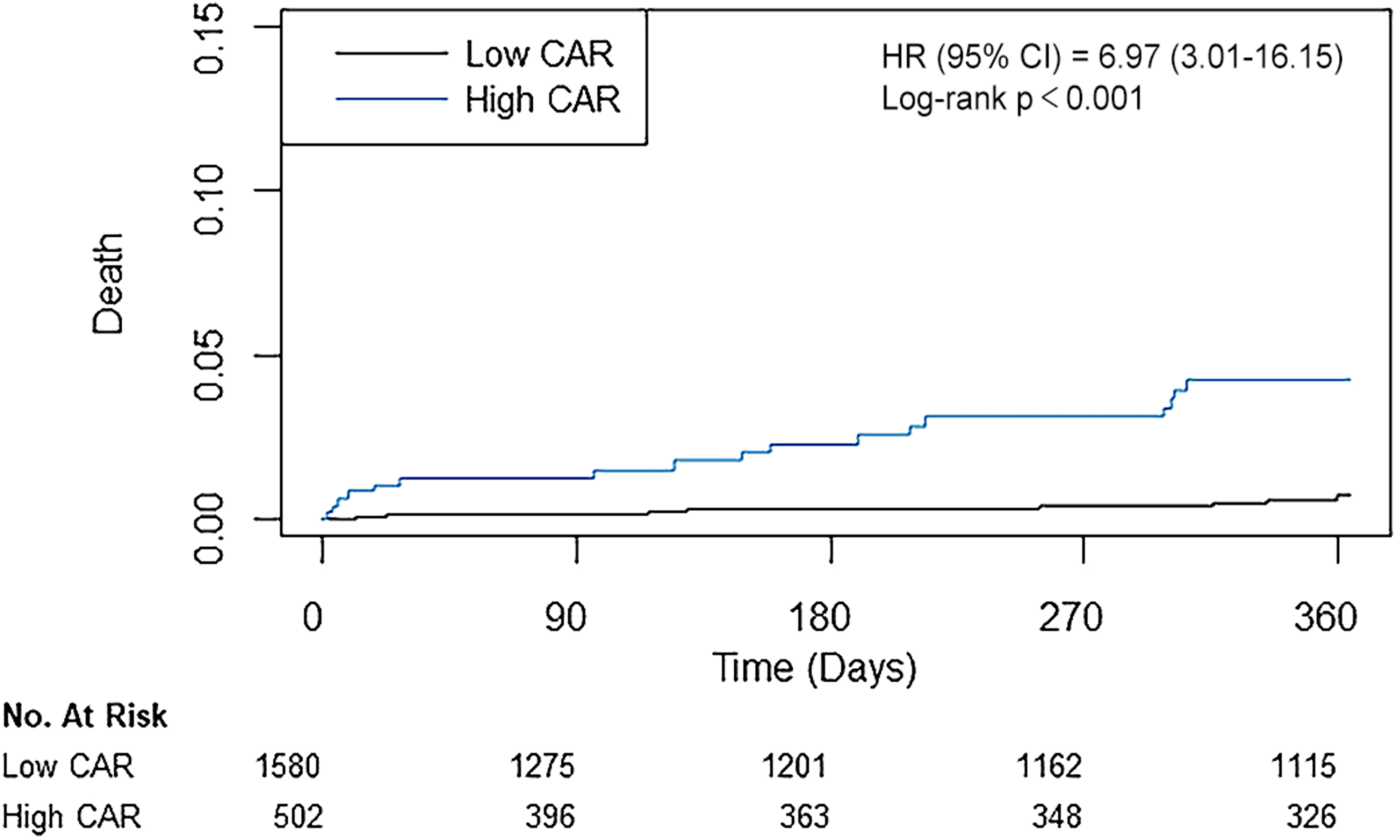
Kaplan–meier curves for 1-year mortality.

According to the calculated cut-off value of CAR, the patients were divided into two groups: 1,580 (75.9%) patients in the low CAR group vs. 502 (24.1%) patients in the high CAR group. The baseline characteristics of patients according to CAR level are presented in Table 2. The median [IQR] value of preoperative CAR was 0.02 [0.09–0.49] in the low CAR group and 3.32 [1.85–7.23] in the high CAR group (*p* < 0.001). Other inflammatory markers were also significantly higher in the high CAR group, whereas albumin and lymphocyte were significantly lower. The high CAR group was older and had a higher frequency of acute myocardial infarction, peripheral arterial occlusive disease, stroke, and chronic kidney disease, while having decreased ejection fraction and a lower history of statin use. After IPW adjustment, the covariates were well balanced between the two groups. The results of Cox regression analyses are presented in Table 3. The unadjusted analysis demonstrated that the high CAR was significantly associated with increased mortality risk regardless of follow-up period (0.5% vs. 3.4%; HR, 6.97; 95% CI, 3.01–16.15; *p* < 0.001 for 1-year mortality, 1.5% vs. 5.0%; HR, 3.57; 95% CI, 2.04–6.25; *p* < 0.001 for 3-year mortality, 2.6% vs. 6.4%; HR, 2.80; 95% CI, 1.76–4.44; *p* < 0.001 for 5-year mortality), but 1-year MACCE was not significantly associated with high CAR (5.4% vs. 6.2%; HR, 1.23; 95% CI, 0.81–1.85; *p* = 0.33). This trend persisted after adjustment with IPW (HR, 5.01; 95% CI, 2.01–12.50; *p* < 0.001 for 1-year mortality, HR, 2.44; 95% CI, 1.31–4.54; *p* = 0.01 for 3-year mortality, HR, 1.91; 95% CI, 1.15–3.21; *p* = 0.01 for 5-year mortality, HR, 1.11; 95% CI, 0.69–1.78; *p* = 0.66 for 1-year MACCE). The ROC curves of CAR for 1-year mortality and MACCE are presented in Figure 1B. The AUC values for predicting 1-year mortality and MACCE were 0.767 and 0.515, respectively. The Kaplan-Meier curves for 1-year mortality stratified by CAR group are presented in Figure 2.

**Table 2:** Baseline characteristics according to the estimated cut-off point of C-reactive protein/albumin ratio >1.326.

	Low group (*N* = 1,580)	High group (*N* = 502)	*p-*value	ASD before IPW	ASD after IPW
C-reactive protein/albumin ratio	0.20 (0.09–0.49)	3.32 (1.85–7.23)	<0.001		
Neutrophil/lymphocyte ratio	1.86 (1.39–2.55)	2.42 (1.77–3.33)	<0.001		
Platelet/lymphocyte ratio	6.67 (5.28–8.63)	8.63 (6.27–11.46)	<0.001		
C-reactive protein, mg/l	0.8 (0.4–2.0)	13.9 (7.8–28.1)	<0.001		
Albumin, g/dl	4.3 (4.1–4.5)	4.0 (3.7–4.3)	<0.001		
Neutrophil	58.1 (51.1–64.4)	62.1 (55.5–68.3)	<0.001		
Lymphocyte	31.1 (25.2–36.7)	25.4 (20.4–31.8)	<0.001		
Platelet, K/mcL	207 (176–244)	215 (174–257)	0.01		
Age, years	62.8 (±10.0)	64.7 (±10.0)	<0.001	18.4	2.3
Male	1,242 (78.6)	379 (75.5)	0.16	7.4	6.3
Smoking	405 (25.6)	145 (28.9)	0.17	7.3	3
Body mass index	24.7 (±3.1)	24.5 (±2.9)	0.32	5.2	1.8
Hypertension	1,255 (79.4)	398 (79.3)	0.99	0.4	2.4
Diabetes	706 (44.7)	240 (47.8)	0.24	6.3	1.7
Old myocardial infarction	134 (8.5)	52 (10.4)	0.23	6.4	3.9
Acute myocardial infarction	115 (7.3)	110 (21.9)	<0.001	42.4	9.4
Ejection fraction	58.0 (±11.9)	53.4 (±13.4)	<0.001	36.2	7.1
Previous coronary intervention					
Percutaneous intervention	288 (18.2)	73 (14.5)	0.07	10	1.8
Bypass grafting	6 (0.4)	1 (0.2)	0.87	3.4	1
Previous disease					
Peripheral arterial occlusive disease	68 (4.3)	40 (8.0)	0.002	15.3	2.1
Chronic obstructive pulmonary disease	17 (1.1)	12 (2.4)	0.05	10.1	3.2
Stroke	185 (11.7)	78 (15.5)	0.03	11.2	1.4
Chronic kidney disease	65 (4.1)	52 (10.4)	<0.001	24.3	4.2
Dialysis	29 (1.8)	33 (6.6)	<0.001	23.8	2.7
Heart failure	21 (1.3)	8 (1.6)	0.82	2.2	2.3
Valvular disease	9 (0.6)	1 (0.2)	0.5	6	1.3
Aortic disease	8 (0.5)	7 (1.4)	0.08	9.2	2.6
Drug use					
Statin	849 (53.7)	226 (45.0)	0.001	17.5	2
Antiplatelet	1,485 (94.0)	476 (94.8)	0.56	3.6	3.4
Renin-angiotensin-aldosterone system inhibitor	497 (31.5)	163 (32.5)	0.71	2.2	4.8
Beta blocker	546 (34.6)	149 (29.7)	0.05	10.5	1.5
Calcium channel blocker	492 (31.1)	160 (31.9)	0.8	1.6	2.7
Operative variables					
Urgency operation	35 (2.2)	15 (3.0)	0.41	4.9	2.5
Operative duration, min	270.2 (±69.8)	266.6 (±65.1)	0.31	5.3	1.9
Red blood cell transfusion, pack	2.1 (±1.5)	2.4 (±1.6)	0.001	16.5	5.3

Values are *n* (%) or mean (±SD)/median (interquartile range).

**Table 3:** Risk of adverse events according to the estimated cut-off point of C-reactive protein/albumin ratio >1.326.

	Low group (*N* = 1,580)	High group (*N* = 502)	Unaddjusted HR (95% CI)	*p-*value	IPW adjusted HR	*p-*value
1-year mortality	8 (0.5)	17 (3.4)	6.97 (3.01–16.15)	<0.001	5.01 (2.01–12.5)	<0.001
1-year MACCE	85 (5.4)	31 (6.2)	1.23 (0.81–1.85)	0.33	1.11 (0.69–1.78)	0.66
1-year mortality	8 (0.5)	17 (3.4)				
Graft failure	28 (1.8)	3 (0.6)				
Percutaneous coronary intervention	20 (1.3)	4 (0.8)				
Myocardial infarction	9 (0.6)	5 (1.0)				
Stroke	33 (2.1)	6 (1.2)				
3-year mortality	24 (1.5)	25 (5.0)	3.57 (2.04–6.25)	<0.001	2.44 (1.31–4.54)	0.01
5-year mortality	41 (2.6)	32 (6.4)	2.80 (1.76–4.44)	<0.001	1.91 (1.15–3.21)	0.01

CI, confidence interval; HR, hazard ratio; IPW, inverse probability of weighting; MACCE, major adverse cardio and cerebrovascular events.

## Discussion

In this retrospective study, we investigated the relationship between preoperative CAR and 1-year mortality in patients undergoing OPCAB. We found that the incidence of 1-year mortality was higher in the high CAR group, and an elevated CAR during preoperative period was significantly associated with increased mortality risk after OPCAB. Additionally, CAR demonstrated superior predictive accuracy compared to conventional inflammatory markers, indicating that it could be a feasible prognostic marker in cardiac surgery.

In recent years, the role of inflammation in the development of CAD has been extensively studied and attracted a great deal of attention. Inflammatory processes are not only responsible for the initiation and progression of atherosclerosis, but also contribute significantly to the precipitation of acute thrombotic complications (20). Accumulating data indicate that elevated inflammatory markers, such as CRP, NLR, and PLR, have prognostic value for cardiovascular outcomes in patients with CAD (21–23). Thus, inflammation can serve as a potential prognostic factor in CAD, providing valuable clinical information with considerable practical usefulness. However, the usefulness of inflammatory status alone for predicting prognosis may be limited, as it does not account for nutritional status associated with poor clinical outcomes.

CAR is a newly developed marker that provides insight into both the inflammation and nutritional status of patients. Inflammation and malnutrition are closely related, both leading to adverse outcomes including cardiovascular events (24). Inflammation can cause malnutrition, which in turn can negatively impact the management of inflammation (25). It has been reported that an elevated CAR was independently associated with stent restenosis, acute kidney injury, and higher burden of coronary thrombus in acute coronary syndrome (16, 26, 27). Also, CAR presented a higher accuracy for the prediction of outcomes than either of the individual markers (28, 29). To evaluate the association between mortality and CAR in patients undergoing CABG, we hypothesized that the predictive value of CAR would be more evident in OPCAB, a procedure that involves less inflammation compared to on-pump CABG without the use of cardiopulmonary bypass (30, 31). In this study, we found that elevated level of preoperative CAR was also associated with a higher risk of death and had better prognostic value than traditional inflammatory markers in OPCAB patients.

Although previous studies have demonstrated that inflammatory markers are linked to an increased risk of MACCE (32), our study did not find a significant association between the CAR and MACCE. A potential explanation for the lack of significance is that the incidence of MACCE within our study cohort may have been too low to detect a statistically significant association. Specifically, the overall incidence of MACCE in this study was approximately 5.6% (116 of 1,580) within the first year of follow-up, suggesting that our study may not have had sufficient statistical power to detect a significant association. The relationship between CAR and MACCE could be nuanced and may extend over the long term. Therefore, a longer follow-up duration may be necessary to capture the full spectrum of the effect of CAR on MACCE. The relatively short follow-up duration in our study might not fully capture the potential impact of CAR on the occurrence of MACCE, especially if the association is gradual or cumulative over time. Furthermore, it is crucial to acknowledge that the relationship between inflammatory markers and MACCE is complex and not yet fully understood. Our study explored CAR as a potential predictor, but other factors such as surgical complexity and post-operative complications may have exerted a more substantial influence on MACCE risk in surgical patients (33). These factors could act as confounders, potentially overshadowing the association between CAR and MACCE. Given the complexity of these relationships, further investigation is warranted to better understand the precise role of CAR in predicting the risk of MACCE after Off-Pump Coronary Artery Bypass (OPCAB). Future studies could explore extended follow-up periods, incorporate a more comprehensive set of confounding variables, and consider additional inflammatory markers to elucidate the intricate interplay between inflammation, CAR, and MACCE in the context of cardiac surgery. While our study did not reveal a significant association between CAR and MACCE within the observed timeframe, it serves as a valuable contribution to the ongoing discourse surrounding inflammatory markers and cardiovascular outcomes in the context of OPCAB. Continued research efforts are essential for refining risk prediction models and informing clinical decision-making in cardiac surgery patients.

There are several limitations that should be considered when interpreting the results of this study. First, this was a retrospective study conducted at a single center, so our results may lack causality. And it may not be applicable to other patients treated with different perioperative management or surgical techniques. While we controlled for known confounding factors, there may still be unidentified confounding factors that could affect the observed associations. By including only the initial operation, there may be a potential impact on biases, such as selection bias or survival-ship bias. Additionally, the low incidence of 1-year mortality (1.2%) may cause bias or lead to underpowered analyses. Second, this study only considered CAR values during the preoperative period, so it would be valuable to conduct further research that assess changes in CAR during the perioperative period for better understanding of prognosis. Furthermore, there is a potential limitation associated with using blood laboratory tests within six months before surgery, particularly in capturing dynamic changes in acute phase reactants. Third, our study included only the patients undergoing OPCAB without cardiopulmonary bypass. On-pump CABG is related to a stronger inflammatory reaction than OPCAB. Therefore, the CAR may have different effects on mortality in patients undergoing on-pump CABG or other cardiac surgeries using cardiopulmonary bypass. Lastly, whether CAR is a modifiable risk factor in OPCAB is indeterminate in this study. Future studies are required to determine whether preoperative use of anti-inflammatory drugs or albumin correction can improve postoperative outcomes. Nevertheless, our study is noteworthy in that it was the first study to reveal the relationship between preoperative CAR and mortality in patients undergoing OPCAB. CAR may be a useful tool for risk stratification of OPCAB patients with broad clinical applications due to its ease of access.

## Conclusion

In this study, we demonstrated that preoperative CAR was associated with 1-year mortality following OPCAB. CAR may be used as a valuable biomarker to predict mortality in patients undergoing OPCAB with superior predictive power than previous inflammatory markers. Further prospective studies are necessary to validate our findings.

## Data Availability

The raw data supporting the conclusions of this article will be made available by the authors, without undue reservation.
